# Supplementary material for: Light-driven peristaltic pumping by an actuating splay-bend strip
Source: Nat Commun. 2023 Apr 4;14:1877. doi: 10.1038/s41467-023-37445-5 (PMC10073117; doi:10.1038/s41467-023-37445-5)
Supplement: Supplementary file 1 — Supplementary Information [file 41467_2023_37445_MOESM1_ESM.pdf]

Supplementary information
Light-driven peristaltic pumping by an actuating splay-bend strip

Klaudia Dradrach[1,2*], Michał Zmyślony[1], Zixuan Deng[3], Arri Priimagi[3], John Biggins[1†] & Piotr Wasylczyk[2]

[1]Department of Engineering, University of Cambridge, United Kingdom
[2]Faculty of Physics, University of Warsaw, Poland
[3]Faculty of Engineering and Natural Sciences, Tampere University, Finland

Contents

Experimental supplementary information **2**
- Thermal imaging . . . 2
- Elastic moduli of planar, homeotropic, and splay films . . . 2
- Thermal strain of a planarly aligned LCG strip . . . 2
- Spontaneous curvature of a splay-aligned LCG strip . . . 2

Theoretical supplementary information **6**
- Numerical comparison of the deformation for different penalising energies . . . 6
- Obtaining the analytical solution for the bump shape . . . 6
- Deformations for a rectangular stimulus profile . . . 7
  - Strong response regime . . . 7
  - Intermediate response regime . . . 7
  - Weak response regime . . . 8
- Volumes of the bump for all regimes . . . 8
- Volume dependence on the product of width and curvature . . . 8

*kd498@cam.ac.uk
†jsb56@cam.ac.uk

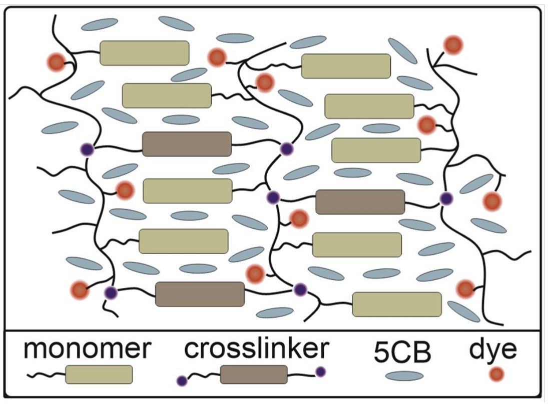

Figure S1: A scheme of the molecular structure of the LCG.

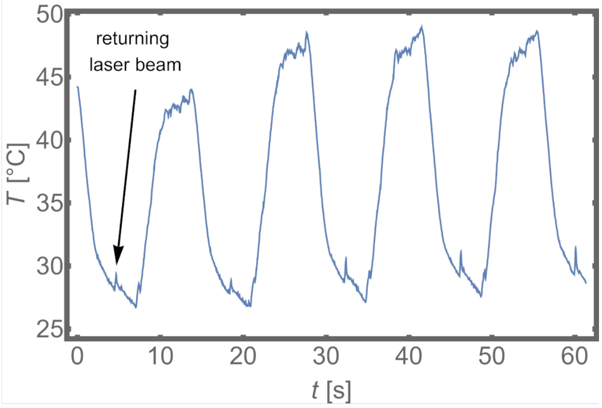

Figure S2: Temperature profile of a small part of the strip. A temperature elevation while liquid pumping (slow scanning) and a beam return (fast scanning) are depicted. The peak intensity of the light was 12.5 W/cm^2.

Experimental supplementary information

Supplementary data provided in this section was collected for strips cut manually from free-standing LCG films. The author's understanding of the molecular structure of this very LCG is presented in Fig. S1.

Thermal imaging

The temperature distribution was measured with a thermal imaging camera (FLIR T420bx) during laser scanning with a scanning speed of 1.2 mm/s. A typical temperature profile during scanning is presented in Fig. S2, where a large periodic increase in temperature can be observed during scanning, and another smaller one corresponds to the fast return of the scanning beam. The increase in temperature during the return was in range 1.5–3.4 °C for peak intensities of 12.5–17.0 W/cm^2. The measured data allowed us to estimate the maximal working temperatures to be 47.5–58.3 °C for peak light intensities 15.5–21.3 W/cm^2, where the beam was circular and had FWHM of 1.7 mm.

Elastic moduli of planar, homeotropic, and splay films

The elastic moduli for LCGs greatly depend on the alignment and the direction of strain in relation to it. There was a slight variation between different samples in the strain-stress measurements, and the results are presented in Fig. S3.

Thermal strain of a planarly aligned LCG strip

Contraction parallel to the director in the sample was measured during microscopic observation as described in Methods in the main text. Collected data for four samples for both heating and cooling is shown in Fig. S4.

Spontaneous curvature of a splay-aligned LCG strip

Spontaneous curvature of the LCG strip can be easily measured by measuring the radius of curvature of a strip in different temperatures (Fig. S5). The curvatures of a 50 µm thick LCG strip for temperatures in the range of 23–98 °C are shown in Fig. S6. Due to the strip being polymerised at 40 °C, the strip was flat at this temperature, and below this temperature, the curvatures were negative, while above it, they were positive. However, this makes no difference for the theory, which only considers the second spatial derivative of the curvature and is entirely insensitive to constant additional factors. Therefore, the theory and the plots in Fig. 3 and Fig. 4 in the main text are zeroed at room temperature for better readability.

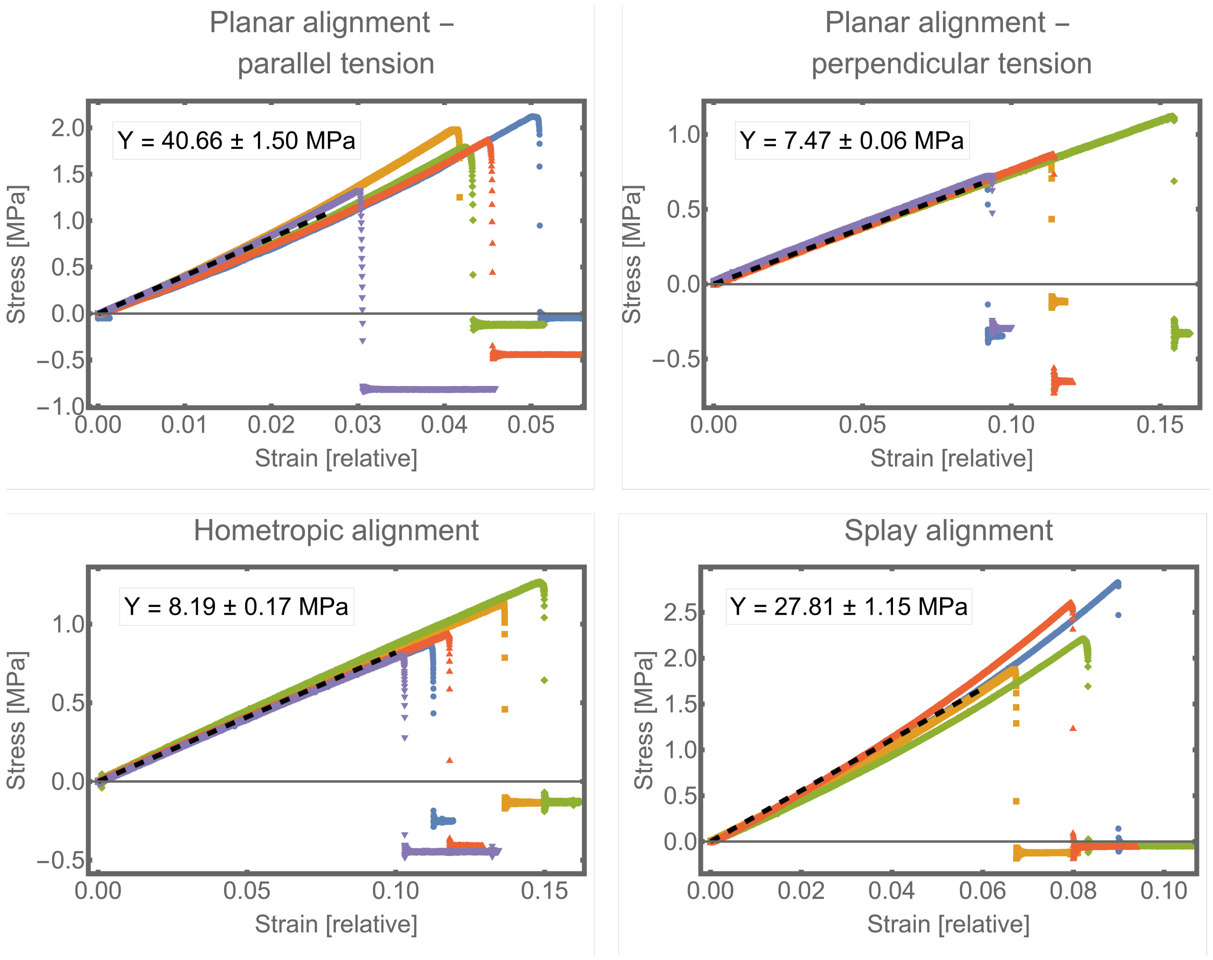

Figure S3: Tensile testing results for different alignments: longitudinally and transversely stretched planarly aligned LCG samples, the homeotropically aligned strip perpendicular to the director, and for splay-aligned samples along the planar part of the director. The panel inset in the plot gives the mean Young's modulus and its standard error, and the black dashed lines use this mean value and are plotted over the strain ranges used for fitting. Different colours show results for different samples.

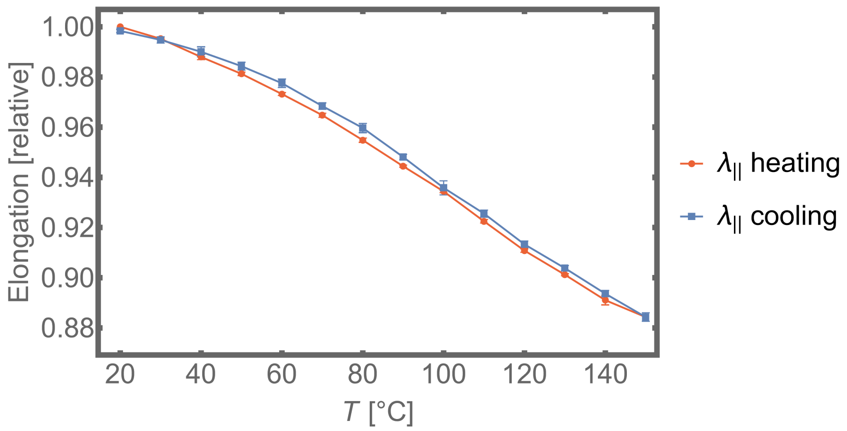

Figure S4: Relative thermal contraction of a planarly aligned LCG strip, parallel to the director for both heating and cooling. The error bars represent the standard error of the mean of the used samples.

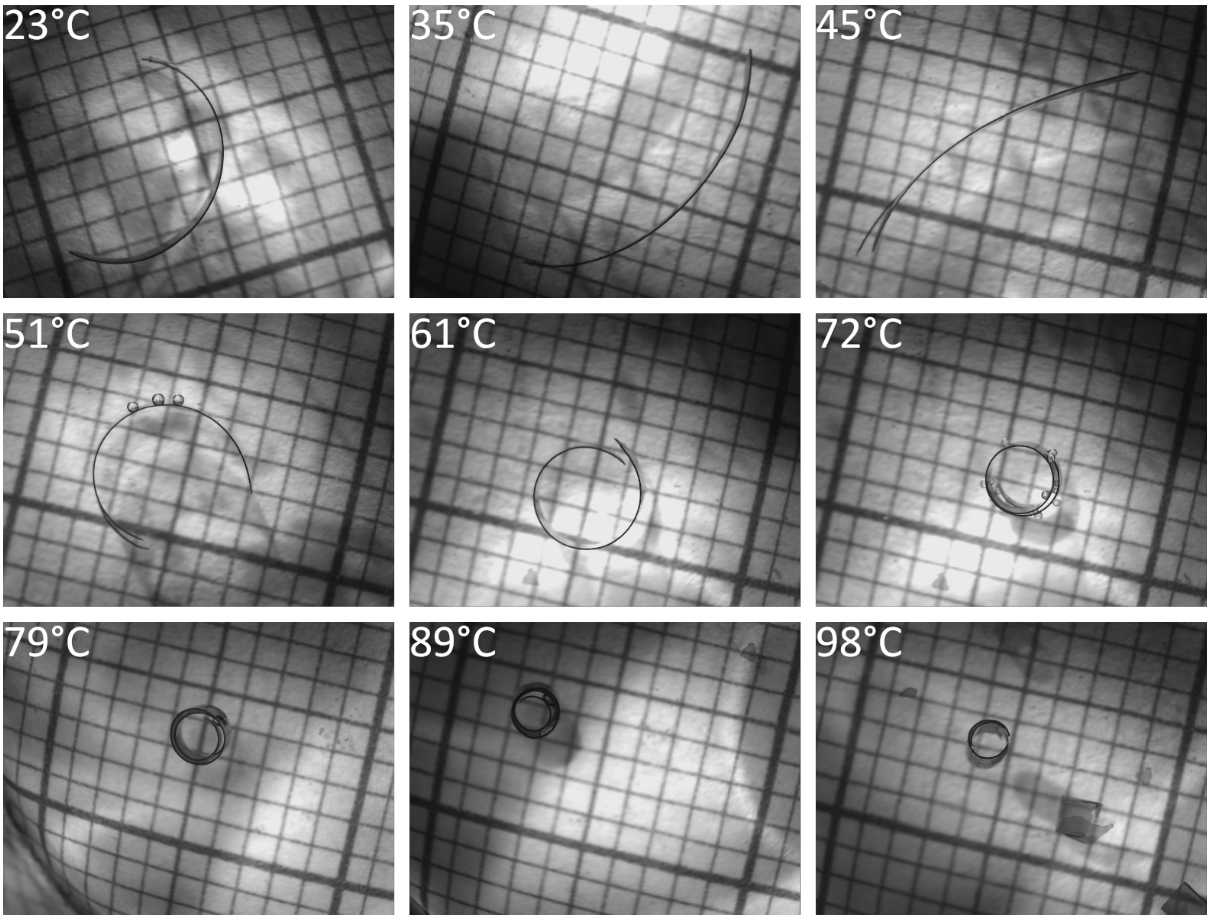

Figure S5: Photos showing the curving of the LCG strip in different temperatures. The checkered background has a spacing of 1 mm.

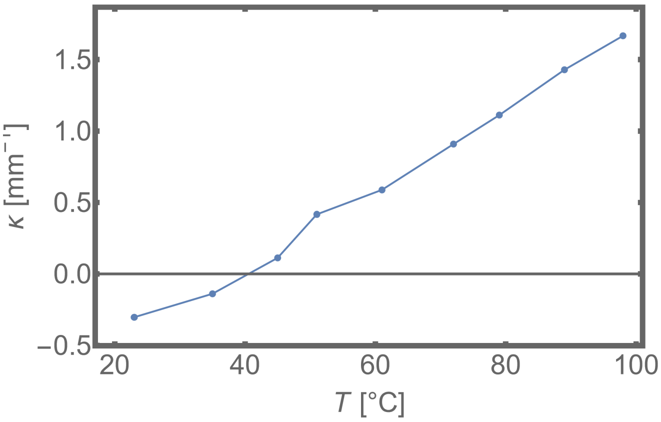

Figure S6: The spontaneous curvature versus temperature of an LCG strip (9 mm x 2 mm x 50 μm) homogeneously heated in a water container on a hot plate. Photos were taken 15–20 min after setting the temperature (ATP DT-612 Thermometer was used to measure temperature).

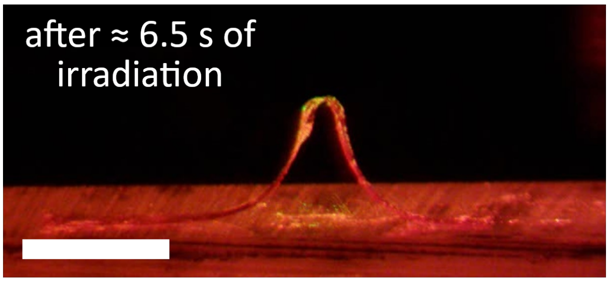

Figure S7: An LCG strip (size: 20 mm x 2 mm x 50 µm) was irradiated with a rectangular beam (xFWHM: 0.447 cm, yFWHM: 0.27 cm) with peak intensity $I_m = 2.46\,\mathrm{W/cm^2}$. The strip was placed on a flat PMMA plate on top of glass microspheres (diameter $\sim 20\,\mu\mathrm{m}$) to prevent adhesion. One end was fixed to the plate, while the other was free to allow sliding during the actuation. The scale bar is 5 mm.

Theoretical supplementary information

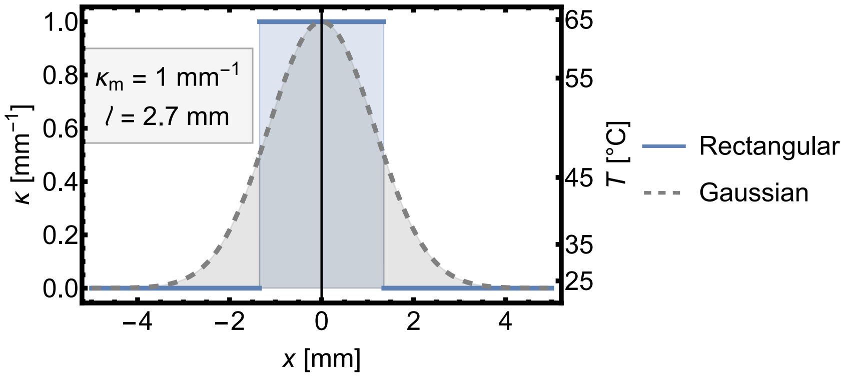

Figure S8: Comparison of the rectangular and Gaussian curvature profiles for the same values of maximal curvatures and lengths.

Numerical comparison of the deformation for different penalising energies

Including the effect of gravity leads to total strip energy:

$$E_{\mathrm{tot}} = \frac{Dw}{2} \int_0^L \left(y''(x) - \overline{\kappa}(x)\right)^2 \, dx + 2\gamma \int_0^L y(x) \, dx + \frac{w\rho g}{2} \int_0^L y^2(x) dx. \tag{1}$$

In Fig. S9, we present numerical minimisations of this energy for three cases — surface tension only, gravity only, and surface tension + gravity — using characteristic strip parameters from our main manuscript. We observe that including gravity makes a negligible difference, confirming that it may be ignored in our system.

Obtaining the analytical solution for the bump shape

In order to obtain the formula for the general bump deformation, we start with the energy functional as defined in the main text

$$E = \frac{Dw}{2} \int_0^L \left(y''(x) - \overline{\kappa}(x)\right)^2 \, dx + 2\gamma \int_0^L y(x) \, dx. \tag{2}$$

This functional is dependent on the deformation via the shape function $y(x)$. When we variationally minimise the energy over possible configurations, we find that the resulting profile satisfies the following differential equation

$$y^{(4)}(x) - \overline{\kappa}''(x) = -\frac{\gamma}{Dw}, \tag{3}$$

which, after being integrated four times, gives us the general form of the bump deformation as

$$y_b(x) = c_0 + c_1 x + c_2 x^2 + c_3 x^3 - \frac{x^4 \gamma}{12 Dw} + \overline{y}(x). \tag{4}$$

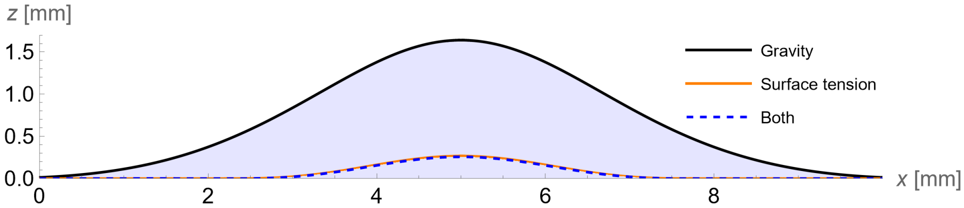

Figure S9: Numerical comparison of deformations for different penalising energies. The strip had w = 2 mm and t = 50 µm, and the stimulus was Gaussian with κ_m = 0.96 mm^{-1} and l = 2.7 mm. The liquid underneath the strip was taken to be water.

In order to obtain the differential equation, we have integrated the energy by parts twice, giving us two boundary conditions for the continuity of the height and the gradient. By solving these boundary equations, we get the shape of the strip as a function of the contacting positions x_1 and x_2. As the deformation is localised, these positions can not be infinitely far from the centre, and in order to find them, we adopt a piece-wise solution, in which the strip is contacting the surface for $x < x_1$ or $x > x_2$ where $x_1 < x_2$. After supplying this form of the solution to the Eq. 2 and minimising the energy over the values of x_1 and x_2 we fix their values and find the whole deformation.

Deformations for a rectangular stimulus profile

Strong response regime

As the Eq. 4 contains the second integral of the preferred curvature, we first calculate it for the rectangular stimulus, keeping in mind that any polynomial terms up to the third order can be absorbed into constants of integration. We choose the second integral to be symmetric around $x = 0$, as due to the symmetry of the stimulus, the deformation will reflect this symmetry, and then the integral is

$$\overline{y}^{\mathrm{R}}(x) = -\kappa_m l^2 \begin{cases} \frac{1}{8} + \frac{|x|^2}{2l^2} & |x| \leq l/2, \\ \frac{|x|}{2l} & |x| > l/2, \end{cases} \tag{5}$$

We then supply it to the Eq. 4 and solve the boundary conditions in order to find the values of all the remaining constants. Due to the symmetry of the problem, we can eliminate all the constants of integration for odd powers of x and look for a solution in which $-x_1 = x_2 > l/2$. After finding the values of the constants of integration, we retrieve the solution from the main text

$$y_s^{\mathrm{R}}(x) = \frac{3}{32}\left(\kappa_m l\right)^{4/3}\lambda + \frac{3}{4}\frac{\left(\kappa_m l\right)^{2/3}}{\lambda}x^2 + \frac{x^4}{2\lambda^3} - \kappa_m l^2 \begin{cases} \frac{1}{8} + \frac{x^2}{2l^2} & |x| \leq l/2, \\ \frac{|x|}{2l} & |x| > l/2, \end{cases} \tag{6}$$

$$x_2 = -x_1 = \frac{1}{2}\left(\kappa_m l\right)^{1/3}\lambda, \tag{7}$$

where we have named it differently to mark that this is a strong response solution.

The height at the centre of the strip is then

$$y_s^{\mathrm{R}}(x) = \frac{1}{32}\kappa_m l\left(3\left(\kappa_m l\right)^{1/3}\lambda - 4l\right), \tag{8}$$

which gives the threshold curvature for the onset of the strong response as $\kappa_m^* = \frac{4^3}{3^3}\frac{l^2}{\lambda^3}$. For the curvatures below this threshold, the height of the strip would become negative.

Intermediate response regime

In the new problem, the height and its gradient (angle of the slope) are both zero at the centre of the system, but the second derivative is not. We then look for a solution in which $y(0) = y'(0) = 0$ and $y(x_2) = y'(x_2) = y''(x_2) = 0$. We do not need to look for the corresponding solution for x_1 as we will get it by symmetrising the result. The boundary conditions can be reduced to obtain a set of equations

$$\begin{aligned} c_0 &= \frac{1}{8}\kappa_m l^2, \\ c_1 &= \frac{1}{2}\kappa_m l, \\ c_2 &= \frac{\kappa_m l}{2x_2} - \frac{x_2^2}{\lambda^3}, \end{aligned} \qquad \begin{aligned} c_3 &= \frac{4x_2}{3\lambda^3} - \frac{\kappa_m l}{6x_2^2}, \\ \frac{3}{4}\lambda^3\kappa_m l^2 &= x_2^4 + x_2\lambda^3\kappa_m l, \end{aligned} \tag{9}$$

in order to solve this, we need to find the root of a fourth-order polynomial. This causes the full solution to be very complicated, so we will refrain from showing it as it gives no additional insight. However, if we keep decreasing the curvature, we will eventually cause the curvature of the strip at the centre to become zero, causing the boundary conditions at the centre to be the same as at the edges in the strong response case. This will cause the separation of the deformation into two, with both the deformations centred around respective slopes of the rectangular function.

Weak response regime

In this regime, the curvature is below $\kappa_m^{**} = \left(6\sqrt{3}-9\right)\frac{l^2}{\lambda^3}$ and we look for a solution in which $\xi_R - \Delta\xi < 0 < \xi_R$, where $\xi = x - l/2$ is the position in relation to the slope of the preferred curvature, ξ_R is the right-most boundary position and $\Delta\xi$ is the width of the bump. The profile is then given as

$$y_w^R(\xi) = \frac{1}{864}\lambda^3\kappa_m^2 - \frac{\lambda^{3/2}\kappa_m^{3/2}}{18\sqrt{2}\sqrt[4]{3}}\xi + \frac{\kappa_m}{4}\mathrm{sgn}(\xi)\xi^2 - \frac{\sqrt{2}\sqrt{\kappa_m}}{3^{3/4}\lambda^{3/2}}\xi^3 - \frac{1}{2\lambda^3}\xi^4, \tag{10}$$

$$\xi_R = \frac{1}{6}\sqrt{2\sqrt{3} - 3\lambda^{3/2}\sqrt{\kappa_m}}, \tag{11}$$

$$\Delta\xi = \frac{\lambda^{3/2}\sqrt{\kappa_m}}{\sqrt{2}\sqrt[4]{3}}, \tag{12}$$

which, as we can see, does not depend on the l, and the changing of the curvature only moves the deformation further from the origin.

Volumes of the bump for all regimes

Depending on the regime in which the system is, the formulas for volumes will differ. For the high-curvature regime, the volume is the highest and increases the fastest and is given by

$$V_h^R = \frac{1}{120}w\kappa_m l\left(3(\kappa_m l)^{2/3}\lambda^2 - 5l^2\right). \tag{13}$$

In the intermediate regime,

$$V_i^R = -\frac{1}{24}lw\left(l^2 - 6x_2 l + 6x_2^2\right)\kappa_m - \frac{wx_2^5}{5\lambda^3}, \tag{14}$$

where we once again have not substituted the value of x_2 to improve clarity. It may seem negative, but for the narrow range of κ_m for which this solution is applicable, it remains positive.

Then, when we transition to the low-curvature regime, the volume is given by a simple formula

$$V_l^R = \frac{w\sqrt{\kappa_m^5\lambda^9}}{540\sqrt[4]{12}} = \frac{1}{30}\sqrt{\frac{D^3w^5\kappa_m^5}{\sqrt{27}\gamma^3}}, \tag{15}$$

where we observe no dependence on the length of the stimulus and a simple power law scaling.

As the high-curvature regime has efficient pumping action, we introduce threshold volume as the volume of transition from intermediate to strong response regime. This volume is given as

$$V^{R*} = \frac{8}{5\times 3^5}\frac{l^5 w}{\lambda^3} = \frac{4}{5\times 3^6}\frac{l^5\gamma}{D}, \tag{16}$$

and it was used to find the threshold scaling law for the Gaussian stimulus, for which l was the FWHM of the curvature profile.

Volume dependence on the product of width and curvature

In order to prove that the volume of the liquid underneath the strip depends on the product of the curvature and width, we start with rewriting Eq. 3 as

$$wy^{(4)}(x) - w\overline{\kappa}''(x) = -\frac{\gamma}{D}. \tag{17}$$

By introducing a re-scaled height function $\tilde{y}(x) = wy(x)$ and calling the product of $w\overline{\kappa}(x) = \tilde{K}(x)$, we can rewrite the previous equation as

$$\tilde{y}^{(4)}(x) - \tilde{K}''(x) = -\frac{\gamma}{D}, \tag{18}$$

which now shows that $\tilde{y}$ no longer depends on neither w nor $\overline{\kappa}$ separately. Now, we retrieve the volume by integrating $\tilde{y}$ from $-\infty$ to $+\infty$. Therefore, we have shown that the volume only depends on the product of width and curvature.
