# Supplementary material for: Light-driven peristaltic pumping by an actuating splay-bend strip
Source: Nat Commun. 2023 Apr 4;14:1877. doi: 10.1038/s41467-023-37445-5 (PMC10073117; doi:10.1038/s41467-023-37445-5)
Supplement: Supplementary file 3 — Description of Additional Supplementary Files [file 41467_2023_37445_MOESM3_ESM.docx]

**Description of Additional Supplementary Files**

for

**Light-driven peristaltic pumping by an actuating splay-bend strip**

Klaudia Dradrach^1,2∗^, Michał Zmyślony^1^, Zixuan Deng^3^, Arri Priimagi^3^, John Biggins^1†^ & Piotr Wasylczyk^2^

^1^Department of Engineering, University of Cambridge, United Kingdom

^2^Faculty of Physics, University of Warsaw, Poland

^3^Faculty of Engineering and Natural Sciences, Tampere University, Finland

Supplementary Movie 1 – Top-side view of a peristaltic pump transporting water.
In this movie, light-driven peristaltic pumping is demonstrated. The pump (17 mm x 2 mm x 50 μm) was placed on a stiff polymeric plate in order to connect two wells, one filled with water (on the left) and the second empty one. The circular laser beam was scanning the pump with velocity 1.2 mm/s.

Supplementary Movie 2 – Side view of a peristaltic pump: inflow, transport, and outflow of the liquid.
A peristaltic pump (22 mm x 2 mm x 50 μm) was placed between two wells on a polymeric plate and scanned with a laser beam (FWHM 4.45 mm x 2.7 mm). In the first part of the movie, a pick-up process from the first well is shown, followed by the propagation of deformation with a portion of water underneath. Finally, in the movie’s last part, a water deposition in the second well is shown.

Supplementary Movie 3 – Inverted fluid transport
The pump (20 mm x 2 mm x 50 μm) was placed on a stiff polymeric plate and scanned by the laser beam (FWHM 4.45 mm x 2.7 mm). Water transport was observed. During scanning, the polymeric plate was turned upside down, and then the movie of water transportation was recorded. The surface of the polymeric plate was wiped with a cotton bud (0:07–0:09 s) to demonstrate that the pump is on the other surface.

Supplementary Movie 4 – Theoretical deformation of a strip for a variable curvature
A strip is stimulated with a rectangular stimulus of length l = 2.7 mm and preferred curvature κ_m_ in range 0.01–1.5 mm^−1^.
The top half of the movie shows an image of a strip as it actuates with a true aspect ratio, with the red area marking the part with non-zero preferred curvature, whereas the orange part has no preferred curvature. The bottom half of the movie has a variable aspect ratio which is required to see the behaviour of the strip for very weak stimuli. The deformation goes from all three response regimes, starting with the weak response regime, going through the intermediate and stopping at the strong response before going in the reverse order.

Supplementary Movie 5 – Theoretical deformation of a strip for a variable length of the stimulus
In this Movie, the stimulus has constant preferred curvature of κ_m_ = 0.8 mm^−1^ and the length l  varies in the range 0.1–12 mm. Here, conversely to Supplementary Mov. 4, we start in the strong response regime before going through the intermediate into the weak one and back again. We observe that once the strip reaches the weak response, the shape of the deformation no longer changes but only travels further from the origin.

Supplementary Movie 6 – Reversible water transport
A peristaltic pump (22 mm x 3 mm x 50 μm), placed between two wells on a polymeric plate was stimulated with a beam of length l = 2.7 mm. The scanning direction for the first few scans is from the first to the second well (from left to right). Then scanning direction is reversed, and the pump transports water from the second to the first well (from right to left). In the end, the scanning direction is reversed one more time (from left to right).

Supplementary Movie 7 – Cross-shaped actuator

A 50 μm-thick cross-shaped LCG actuator (with arms’ length 22 mm and width 4 mm) was placed on a polymeric plate with four wells (Fig. 5d(i) in the main text). Two wells were filled with water. A scanning laser beam (extended by a set of lenses including a cylindrical lens; l = 1.29 mm) was used to actuate the LCG cross. Firstly, two opposite arms of the cross (connecting the first full well and the first empty well) were actuated. Then the plate was rotated, and the other two opposite arms of the cross (connecting the second full well and second empty well) were actuated (Fig. 5d(i) in the main text). Water transportation was observed (Fig. 5d(ii)-(iii) in the main text).
